# A Case of Neuralgia

**Published:** 1848

**Authors:** J. George Osbourne

**Affiliations:** Tippecanoe county, Indiana


### ARTICLE VII.

*A Case of Neuralgia.* By J. GEORGE OSBOURNE, M. D., of Tippecanoe county, Indiana.

During the month of July, 1847, Mrs. G——, aged 48, of spare habit, and an inveterate smoker, was occasionally attacked with paroxysms of pain in the ball of the great toe—health otherwise good. These attacks becoming more frequent and severe, she was induced, about the first of August, to seek medical advice, and I was requested to visit her.

She informed me that she had used, without relief, stimulating embrocations and a blister to the entire toe and dorsum of the foot—meantime, the pain grew more constant and severe, when she was advised by a friend to apply a poultice saturated with the tinc. opii which produced some temporary relief.

I found the toe of the natural size and color, the pain not increased by pressure except at a minute point near the centre of the ball, from which (when pressed upon) intense pain would shoot along the course of the principal nerves of the leg and thigh—appetite good, bowels regular, skin wearing the appearance of health, great restlessness at night, considerable emaciation and debility.

I advised a gentle cathartic, to be followed with carb. ferri and pulv. valerian, each 10 grs. thrice daily, and an opiate at bed time; the bowels to be be kept open with castor oil.

Aug. 5th.—No amendment; prescription continued, with the addition of frictions over the entire limb twice daily.

13th.—Symptoms no better; emaciation progressing rapidly; suspended the use of the medicine, and advised the cold douche to the limb, to be followed with frictions night and morning, and an entire abstinence from the use of tobacco.

20th.—Expressed herself as being much better; rests well at night; advised to continue the abstinence and cold douche.

Sept. 1.—Discharged; cured.

The foregoing case, I think, is strongly illustrative of the injurious effects of tobacco on the nervous system. While the patient continued to smoke, the neuralgic affection continued with increased violence, notwithstanding the free use of stimulating and anodyne applications locally and the internal use of tonics, anodynes, and gentle cathartics.

As soon, however, as entire abstinence from the use of the pipe was enjoined, the violence of the symptoms began to abate, and in about two weeks the patient was discharged, cured.

The question naturally arises, What benefit was derived from the use of the douche? or, was the cure entirely attributable to the abstinence from the use of tobacco?

